# Varying effects of tyrosine kinase inhibitors on platelet function—A need for individualized CML treatment to minimize the risk for hemostatic and thrombotic complications?

**DOI:** 10.1002/cam4.2687

**Published:** 2019-11-12

**Authors:** Suryyani Deb, Niklas Boknäs, Clara Sjöström, Anjana Tharmakulanathan, Kourosh Lotfi, Sofia Ramström

**Affiliations:** ^1^ Department of Biotechnology Maulana Abul Kazam Azad University of Technology West Bengal India; ^2^ Department of Clinical Chemistry and Department of Clinical and Experimental Medicine Linköping University Linköping Sweden; ^3^ Department of Haematology and Department of Clinical and Experimental Medicine Linköping University Linköping Sweden; ^4^ School of Medical Sciences Örebro University Örebro Sweden

**Keywords:** chronic myeloid/myelogenous leukemia, coagulation, hemostasis, personalized medicine, platelets, tyrosine kinase inhibitors

## Abstract

Since their introduction, tyrosine kinase inhibitors (TKIs, eg, imatinib, nilotinib, dasatinib, bosutinib, ponatinib) have revolutionized the treatment of chronic myeloid leukemia (CML). However, long‐term treatment with TKIs is associated with serious adverse events including both bleeding and thromboembolism. Experimental studies have shown that TKIs can cause platelet dysfunction. Herein, we present the first side‐by‐side investigation comparing the effects of currently used TKIs on platelet function and thrombin generation when used in clinically relevant concentrations. A flow cytometry multiparameter protocol was used to study a range of significant platelet activation events (fibrinogen receptor activation, alpha granule, and lysosomal exocytosis, procoagulant membrane exposure, and mitochondrial permeability changes). In addition, thrombin generation was measured in the presence of TKIs to assess the effects on global hemostasis. Results show that dasatinib generally inhibited platelet function, while bosutinib, nilotinib, and ponatinib showed less consistent effects. In addition to these general trends for each TKI, we observed a large degree of interindividual variability in the effects of the different TKIs. Interindividual variation was also observed when blood from CML patients was studied ex vivo with whole blood platelet aggregometry, free oscillation rheometry (FOR), and flow cytometry. Based on the donor responses in the side‐by‐side TKI study, a TKI sensitivity map was developed. We propose that such a sensitivity map could potentially become a valuable tool to help in decision‐making regarding the choice of suitable TKIs for a CML patient with a history of bleeding or atherothrombotic disease.

## Graphical abstract

In this study, we show that different TKIs used for treatment of Chronic Myeloid Leukemia have opposing effects on platelet function, with large inter‐individual differences. Thus, in patients with high risk for hemostatic or thrombotic complications, we suggest that treatment with TKIs should be individualized to minimize risks associated with long‐term treatment.

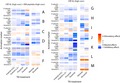

## 1 INTRODUCTION

Tyrosine kinase inhibitors (TKIs) have significantly improved the prognosis for patients with chronic myeloid leukemia (CML).^1^ However, studies have reported on side effects related to treatment with TKIs, including inhibition of platelet function,^2^ cardio‐toxicity,^3^ and congestive heart failure.^4^, ^5^ Because CML patients may need to continue TKI therapy indefinitely, the long‐term safety of each treatment option must be an important consideration. Imatinib, nilotinib, and dasatinib are all approved as first‐line therapy for adult patients.^6^, ^7^, ^8^, ^9^ In imatinib‐resistant patients, second‐generation TKIs like nilotinib and dasatinib as well as bosutinib and ponatinib are considered as second‐ and third‐line therapy.^10^ However, altered hemostasis and gastrointestinal bleeding in dasatinib‐treated patients^11^, ^12^, ^13^ and affected platelet function in ponatinib‐treated patients^14^, ^15^, ^16^ have been reported. Other TKIs such as bosutinib and nilotinib show higher cardiovascular event rates (peripheral arterial occlusive disease, ischemic heart disease, or stroke).^5^, ^17^ Therefore, it is important to understand how TKIs affect the pathophysiological processes that lead to bleeding or thrombosis.

Platelets play a key role in maintaining hemostasis under normal physiological conditions. Their role is to adhere to subendothelial proteins exposed upon vessel wall injury.^18^ Contact with activating substances such as collagen and thrombin results in the formation of a platelet aggregate through fibrinogen binding.^19^, ^20^ However, without reinforcement by a fibrin network, this aggregate (“platelet plug”) will rapidly dissolve. To stabilize the clot, activated platelets become procoagulant by scrambling of membrane to expose negatively charged phosphatidylserine (PS) on their surface.^21^, ^22^ Plasma coagulation factors assembled on PS‐exposing platelet surfaces significantly increase thrombin formation, which in turn induces the formation of a fibrin network to strengthen the clot.^23^, ^24^ Activated platelets also release substances stimulating their neighboring platelets. All of these functions are necessary to stop bleeding. Therefore, any decrease or increase in platelet reactivity may increase the risk of bleeding or thrombus formation, respectively. The clinically used TKIs are designed to have inhibitory effects specific to the Bcr‐Abl kinase, but as mentioned earlier, there are many reports on platelet malfunction or hemostatic alterations by these drugs.^2^, ^5^, ^11^, ^15^, ^16^, ^17^ Both from a clinical and biological perspective, it is important to know how the clinically used TKIs alter normal hemostasis. Whether platelets from every individual under the drug regime will respond to TKIs in the same manner and intensity is another important question to address, as not all patients suffer the same adverse effect from TKIs.

In this side‐by side study, we have performed a comparative analysis of all TKIs currently used in CML therapy, measuring the effects on hemostatic properties such as various aspects of platelet function and thrombin generation. We found general trends in hemostatic alterations, along with large individual variations in response to TKIs. After clinical validation, we suggest that our developed flow cytometry protocol could prove valuable for the clinical management of individual CML patients in order to reduce the risk of thrombotic or hemostatic complications.

## 2 MATERIALS AND METHODS

### 2.1 Materials

The following TKIs were investigated: imatinib and nilotinib (Novartis, Basel, Switzerland), dasatinib (Bristol‐Myers Squibb), ponatinib (ARIAD Pharmaceuticals (Cambridge), and bosutinib (Pfizer). The platelet agonists were cross‐linked collagen‐related peptide (CRP‐XL) with the sequence Gly‐Cys‐Hyp‐(Gly‐Pro‐Hyp)_10_‐Gly‐Cys‐Hyp‐Gly‐NH_2_ and cross‐linked with SPDP (3‐(2pyridyldithio) propionic acid N‐hydroxysuccinimide ester) purchased from Dr Richard Farndale (Cambridge, UK); ADP (adenosine 5ʹ‐diphosphate monopotassium salt dihydrate) from Sigma; protease‐activated receptor1‐activating peptide (PAR1‐AP, sequence SFLLRN) and protease‐activated receptor4‐activating peptide (PAR4‐AP, sequence AYPGKF) from JPT Peptide Technologies GmbH. The HEPES buffer contained 137‐mmol/L NaCl, 2.7‐mmol/L KCl, 1‐mmol/L MgCl_2_, 5.6‐mmol/L glucose, 1 g/L bovine serum albumin, and 20‐mmol/L HEPES (4‐(2‐hydroxyethyl)‐1‐piperazineethanesulfonic acid), pH 7.4. In activation tubes, HEPES buffer with 1.5‐mmol/L calcium (HEPES‐Ca^2+^) was used to enable annexin V binding. HEPES buffer with 10‐mmol/L ethylenediaminetetraacetic acid (EDTA) was used for the EDTA control. All chemicals for the HEPES buffers were of reagent grade and from Sigma. Among flow cytometry markers, DilC_1_(5) (1,1′,3,3,3′,3′‐hexamethylindodicarbocyanine iodide) was from Molecular Probes, CCCP (Carbonyl cyanide 3‐ chlorophenylhydrazone) was from Sigma, and anti‐GPIIb(CD41)‐ECD (clone P2) from Beckman Coulter. All other markers (Annexin V‐V450, anti‐LAMP‐1(CD107a)‐PC7 (clone H4A3), anti‐P‐selectin (CD62P)‐PE (clone AK4), PE‐ and PC7‐isotype control antibodies (Mouse IgG1κ), and PAC‐1‐FITC) were from BD Biosciences. Multiplate^®^aggregometry was performed using the reagents ADP, collagen, and TRAP from the same manufacturer. For thrombin generation, we used the PRP reagent (Thrombinoscope BV) and for free oscillation rheometry (FOR), the ReoTRAP kit (MediRox AB).

### 2.2 Blood collection

Blood was collected via a 21‐gauge needle from healthy volunteers and CML patients with their due consent. The blood collection procedure was approved by the local ethics committee. The only exclusion criterion for participation was that the volunteers were not allowed to have taken any drug known to interfere with platelet function in 14 days before sampling. Blood counts were performed on samples, and all participants were found to have platelet counts in the normal range. The age range was 22‐41 years and 64% were females. For flow cytometry and Multiplate^®^aggregometry, blood was collected into hirudin tubes (final concentration 15 µg/mL, Roche), while citrate tubes (0.105 mol/L tri‐sodium citrate, BD Vacutainer) were used for FOR and thrombin generation assays.

### 2.3 Platelet preparation for flow cytometry

For in vitro experiments, hirudinated whole blood was incubated with different TKIs. The doses of the drugs were chosen from earlier published studies of peak plasma concentrations (*C*_max_) at steady‐state in humans treated with each drug in recommended doses. The concentrations chosen were for dasatinib (100 mg/day) 0.16 μmol/L,^25^, ^26^ for ponatinib (45 mg/day) 0.145 μmol/L,^27^ and for imatinib 4.8 μmol/L^28^, ^29^, ^30^ and 7.5 μmol/L^31^ to reflect both the standard dose (400 mg/day) and the doubled dose (800 mg/day) given to patients who do not respond to standard imatinib treatment. For bosutinib (500 mg/day) and nilotinib (400 mg twice daily), the concentrations were 0.4 μmol/L^32^ and 4.2 μmol/L,^33^, ^34^ respectively. For ex vivo experiments, hirudinated blood was drawn from CML patients.

Drug‐incubated blood (incubation time 10 minutes at room temperature) or patient's blood (3 μL) was added to tubes with platelet agonists and markers (33 μL) which correspond to a 1:12 dilution. ADP, CRP‐XL, PAR1‐AP, and PAR4‐AP were used as agonists to activate platelets in different combinations. The concentrations of agonists were as follows: ADP (10 μmol/L), CRP‐XL (1.2 μg/mL), PAR1‐AP (30 μmol/L), and PAR4‐AP (300 μmol/L). The six color flow cytometry protocol used to study platelet activation markers has been recently described.^35^ Specific settings for this study were as follows: 2.67 μg/mL of Annexin V‐V450 used to detect exposed PS, 0.5 μg/mL of Anti‐LAMP‐1‐PC7 (lysosome‐associated membrane glycoprotein‐1) to detect lysosomal exocytosis, 0.17 μg/mL of Anti‐P‐selectin‐PE to detect alpha‐granule release, 30 nmol/L of DilC_1_(5) to detect mitochondrial membrane potential alterations, 0.56 μg/mL of PAC‐1 FITC (Clone PAC1) to detect the activated conformation of fibrinogen receptor GPIIb/IIIa, and 0.69 μg/mL of Anti‐GPIIb ECD (CD41) to identify platelets and platelet‐derived particles (all final concentrations). CCCP (100 µmol/L) was used as negative control for DilC_1_(5) as it disrupts the mitochondrial membrane.^36^ A tube with HEPES without calcium was used as a negative control for Annexin V. It also contained the PE‐ and PC7‐isotype control antibodies that were used as negative controls for anti‐P‐selectin‐PE and anti‐LAMP‐1‐PC7. As negative control for PAC‐1, EDTA was included in the HEPES buffer to chelate calcium, without which PAC‐1 cannot bind to GPIIb/IIIa.

After addition of the blood to the agonist‐marker reaction mixture, it was incubated at room temperature for 10 minutes. After 10 minutes, the test and control tubes were diluted 1:20 with HEPES with or without calcium, respectively. All tubes were then immediately analyzed on a flow cytometer (Gallios; Beckman Coulter) using the extra wide angle for forward scatter detection.^37^

### 2.4 Thrombin generation

Thrombin generation was monitored using the calibrated automated thrombogram (CAT) method. The experiment was performed in a 96‐well plate. Citrated blood was centrifuged at 140 g, 20°C for 20 minutes to extract platelet‐rich plasma (PRP). The rest of the blood was centrifuged at 1000 g, 20°C for 15 minutes to get platelet‐poor plasma (PPP). The PRP was diluted with PPP to a platelet count of 250 × 10^9^/L and incubated with different TKIs for 10 minutes at room temperature, then activated with CRP‐XL (1.2 μg/mL) at 37°C for another 10 minutes before addition of calcium, fluorogenic substrate, and the PRP reagent (containing 0.5‐pM tissue factor and low amounts of phospholipids). Analysis of thrombin generation was performed on an Ascent FL (Thermo Electron Corporation) with Thrombinoscope software (Thrombinoscope). The analysis program calculates all parameters of the thrombogram and expresses results as nanomolar thrombin with time.^38^

### 2.5 Multiplate aggregometry assay

Platelet aggregation was studied using a Multiplate^®^ analyzer (Roche Diagnostic GmbH) following the instructions from the manufacturer. For this study, venous blood from CML patients was collected in hirudin tubes and allowed to rest for 30 minutes. By that time, 300 µL NaCl was added to the disposable test cells placed in the instrument. Then, 300 µL of hirudinated blood was added to the test cell and incubated for 3 minutes at 37°C under stirring conditions. After that, 20 µL of platelet agonist (collagen, ADP, TRAP, and ASPI, final concentrations 3.2 µg/mL, 6.5 µmol/L, 32 µmol/L, and 0.5 mmol/L, respectively) was added and aggregation measurements started.^38^

### 2.6 Viscoelastic hemostasis assay

Viscoelastic whole blood coagulation measurements were performed by FOR (ReoRox G2, MediRox AB). For this study, citrated venous blood from CML patients was collected. Then, 50‐µL ReoTRAP reagent and 25‐µL 0.5 mol/L CaCl_2_ were mixed with 1‐mL citrated blood by gently pipetting up and down three times using a disposable 1‐mL syringe. The mixed blood (1 mL) was added to the reaction chamber and the software for detection of viscosity and elasticity was initiated.^39^

### 2.7 Presentation of data and Statistical analysis

The data obtained with blood from healthy volunteers with different TKIs were subjected to statistical analysis using GraphPad Prism 5 software (GraphPad Software). As it was technically impossible to measure all TKIs with all agonist combinations in a single experiment, the results were only compared to the corresponding control (blood without TKI addition) for the same run. Data are presented as mean ± standard error of mean (SEM). The effect of the TKI treatments as compared to the corresponding control was compared using ANOVA (with Dunnett's multiple comparison test). The graphical analysis of the platelet TKI sensitivity map was performed using R software (R Foundation for Statistical Computing, Vienna, Austria; http://www.Rproject.org).

## 3 RESULTS

### 3.1 TKIs induce changes in platelet procoagulant activity and mitochondrial membrane potential

**Figure 1:**
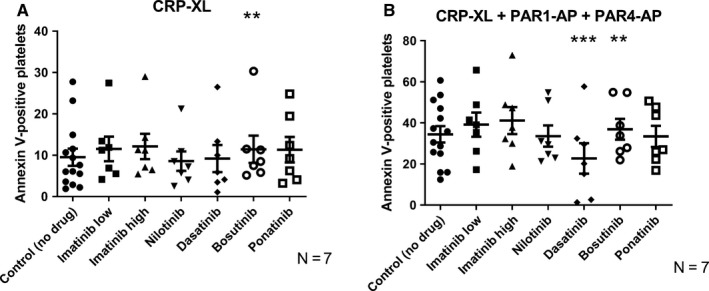
Changes in procoagulant membrane exposure in the presence of TKIs. Platelet phosphatidylserine expression (marker of procoagulant platelets, measured as binding of annexin V) in the presence of TKIs (corresponding to *C*_max_) in vitro in platelets activated with (A) CRP‐XL (1.2 μg/mL) and (B) CRP‐XL (1.2 μg/mL) plus PAR‐APs (PAR1‐AP (30 μmol/L) and PAR4‐AP (300 μmol/L)). Blood was taken from healthy donors (n = 7). The results presented are for the total platelet population. The scatter plots show results for the individual donors, the mean value and standard error of the mean (SEM) are also shown. Paired raw data have been used for ANOVA testing. Stars (*) denote significant differences from control where *P ≤ .05, **P ≤ .01, and ***P ≤ .001

It has been previously demonstrated that a fraction of platelets form a subpopulation of PS‐positive (“procoagulant”) platelets which also release PS‐positive microparticles upon strong activation.^35^, ^40^, ^41^ To investigate whether the TKIs could alter this subpopulation formation, we activated platelets with CRP‐XL alone and in combination with PAR1‐AP and PAR4‐AP. In agreement with previous publications,^35^, ^42^ PAR‐ or ADP‐receptor activation alone showed low potency in stimulating the formation of procoagulant platelets (data not shown). To quantify the extent of platelet PS exposure (critical for binding of coagulation factors and accelerating the coagulation cascade) upon strong stimulation in the presence of TKIs, platelets were stained with Annexin V. As shown in Figure 1, bosutinib generally had a stimulatory effect on Annexin V binding, which was statistically significant when platelets were stimulated with CRP‐XL and CRP‐XL + PAR‐APs, whereas dasatinib had a strong inhibitory effect which was significant when platelets were exposed to a combined stimulus of CRP‐XL and the PAR‐APs.

**Figure 2:**
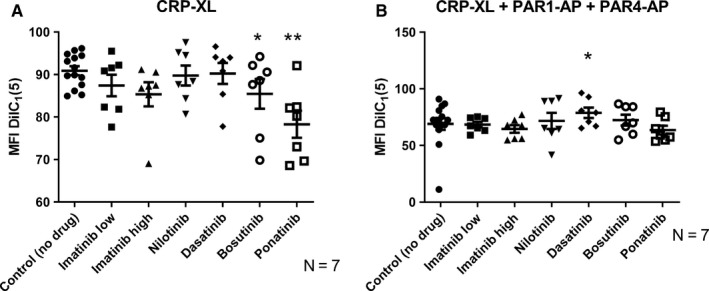
Changes in mitochondrial membrane potential in the presence of TKIs. Alteration of platelet mitochondrial membrane potential (measured by DilC1(5)) by TKIs in platelets activated with (A) CRP‐XL (1.2 μg/mL), (n = 7) and (B) CRP‐XL (1.2 μg/mL) + PAR‐APs (PAR1‐AP (30 μmol/L) and PAR4‐AP (300 μmol/L)), (n = 7). The results presented are for the total platelet population. The scatter plots show results for the individual donors, the mean value and standard error of the mean (SEM) are also shown. Paired raw data have been used for ANOVA testing. Stars (*) denote significant differences from control where *P ≤ .05 and **P ≤ .01

It has been reported that the opening of the mitochondrial permeability transition pore (MPTP) is essential for the formation of procoagulant platelets.^43^ An increase in mitochondrial membrane permeability can be detected as a decrease in DilC_1_(5) fluorescence. Platelets with intact mitochondrial membranes thereby give a higher fluorescence signal than platelets wherein the mitochondrial membranes have become porous (depolarized). As shown in Figure 2, we observed no change in the mitochondrial membrane potential for imatinib‐ or nilotinib‐treated samples. As compared to the sample without drug, ponatinib‐ and bosutinib‐treated platelets showed statistically significant trends toward lower DilC_1_(5) fluorescence and dasatinib‐treated platelets exhibited a statistically significant trend toward higher mitochondrial membrane potential upon stimulation, consistent with stimulatory and inhibitory effects of these drugs, respectively.

### 3.2 Changes in degranulation potential by TKIS

P‐selectin, an adhesion receptor for leukocyte‐platelet interactions, is found in the alpha‐granules of resting platelets. LAMP‐1 is a protein that is situated in the inner membrane of lysosomes. Upon activation, these proteins will become exposed on the platelet surface and can be used as markers for alpha‐granule release and lysosomal exocytosis, respectively.^35^

**Figure 3:**
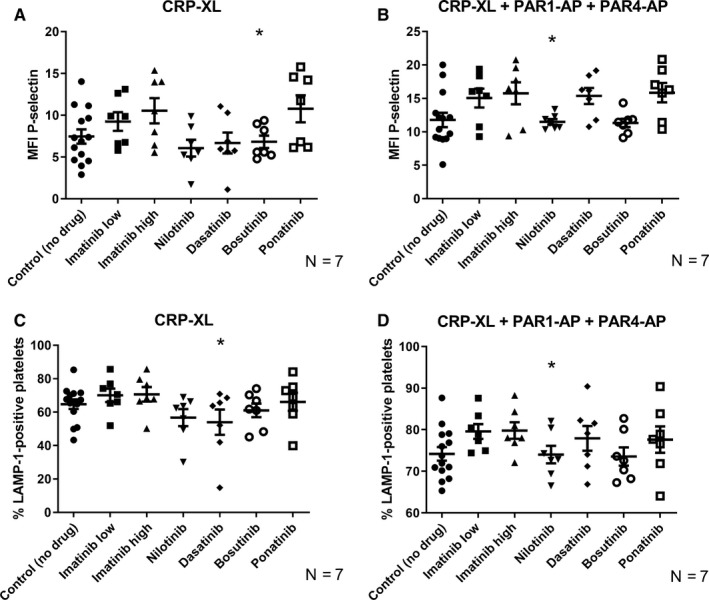
TKI‐induced changes in platelet degranulation. (A‐B) represent exocytosis of alpha‐granules (detected as P‐selectin expression) while (C‐D) represent lysosomal granule release (detected as LAMP‐1 expression) in the presence of different TKIs. In (A and C), platelets were activated with CRP‐XL (1.2 μg/mL) and in (B and D) with CRP‐XL (1.2 μg/mL) + PAR‐APs (PAR1‐AP (30 μmol/L) and PAR4‐AP (300 μmol/L)). The scatter plots show results for the individual donors, the mean value and standard error of the mean (SEM) are also shown. Paired raw data have been used for ANOVA testing. Stars (*) denote significant differences from control where *P < .05

Our results showed that dasatinib treatment resulted in a significant decrease in LAMP‐1 expression after stimulation with CRP‐XL, consistent with an inhibitory effect on granule release (Figure 3). Dasatinib also produced a trend toward lower P‐selectin expression for CRP‐XL, but this trend was not statistically significant. Bosutinib gave a small but significant decrease in platelet P‐selectin expression after stimulation with CRP‐XL (Figure 3A). Nilotinib resulted in a significant decrease in both P‐selectin and LAMP‐1 expressions when activated with CRP‐XL + PAR‐APs (Figure 3B,D). There was no change in granule release (both P‐selectin and LAMP‐1) for any other drug treatment.

### 3.3 Changes in fibrinogen receptor activation by TKIS

**Figure 4:**
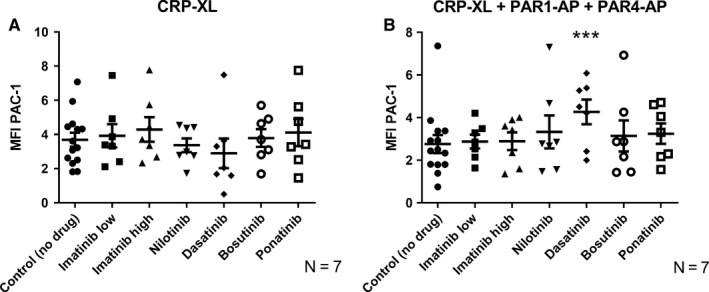
TKI‐induced changes in platelet fibrinogen receptor activation. Active fibrinogen receptor conformation (detected as PAC‐1 binding) in (A) platelets activated with CRP‐XL (1.2 μg/mL) (B) platelets activated with CRP‐XL (1.2 μg/mL) + PAR‐APs (PAR1‐ (30 μmol/L) and PAR4‐ AP (300 μmol/L)). The scatter plots show results for the individual donors, the mean value and standard error of the mean (SEM) are also shown. Paired raw data have been used for ANOVA testing. Stars (*) denote significant differences from control where *P ≤ .05, **P ≤ .01, and ***P ≤ .001

Platelet activation leads to a conformational change in the surface receptor GPIIb/IIIa (detected by the monoclonal PAC‐1 antibody),^44^ which enables platelets to bind fibrinogen and form a platelet aggregate. As shown in Figure 4, in vitro dasatinib treatment resulted in a nonsignificant trend toward decreased expression of the active fibrinogen receptor after activation with CRP‐XL. A significant increase in activated GPIIb/IIIa was observed with dasatinib treatment when platelets were activated with CRP‐XL + PAR‐APs. This increase in PAC‐1 binding can arguably be ascribed to the inhibition of procoagulant platelet formation observed with dasatinib treatment, as procoagulant platelets have reduced PAC‐1 binding (discussed more in detail in the discussion).

### 3.4 TKI‐induced changes in thrombin generation

Thrombin generation in PRP can be used to evaluate the contribution of platelets to thrombin formation.^38^, ^45^ In the CAT assay, different parameters can be extracted to describe the kinetics of thrombin generation, for example, lag time, time to peak, peak height, and ETP (endogenous thrombin potential). In general, bosutinib treatment resulted in a small but significant increase in the lag time (which is the time between tissue factor addition and the start of thrombin generation) (Figure S6A). For the composite variable of ETP, bosutinib‐treated blood showed a consistent and significant increase in ETP (Figure S6C). There were no significant changes observed for any other drugs.

### 3.5 Effects of TKIS on platelet activation with ADP and PAR‐APS

**Figure 5:**
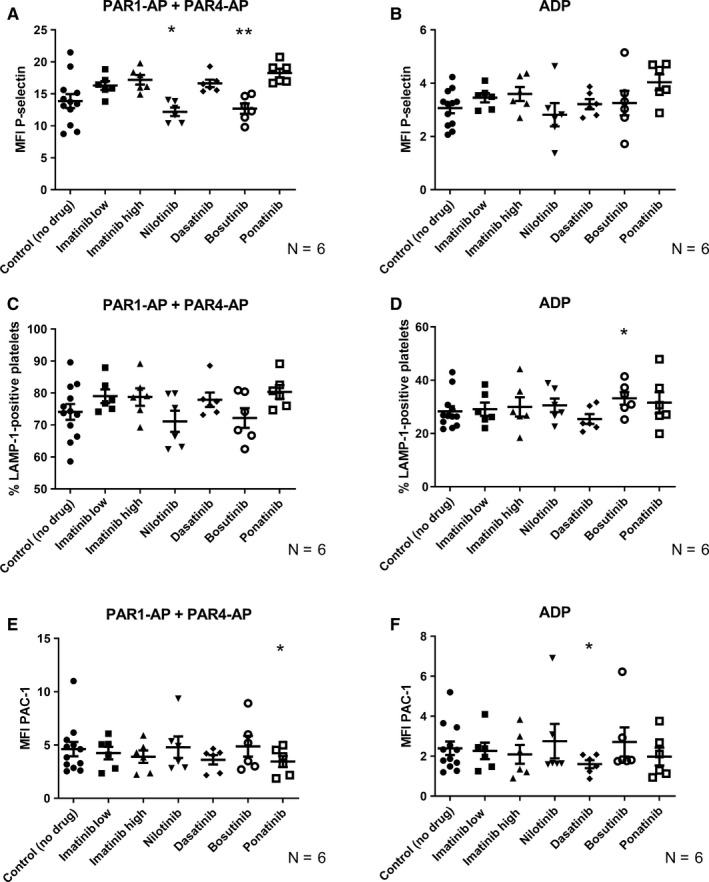
Effect of TKIs on platelet activation with PAR‐APs and ADP. (A‐B) Alpha‐granule release (detected as P‐selectin exposure) (C‐D). Lysosomal exocytosis (detected as LAMP‐1 exposure) and (E‐F) active fibrinogen receptor conformation (detected as PAC‐1 binding) in platelets activated with PAR‐APs (PAR1‐AP (30 μmol/L) and PAR4‐AP (300 μmol/L)) (A, C, E) or ADP (10 μmol/L) (B, D, F). The results presented are for the total platelet population. The scatter plots show results for the individual donors, the mean value and standard error of the mean (SEM) are also shown. Paired raw data have been used for ANOVA testing. Stars (*) denote significant differences from control where *P < .05 and **P < .01, where n = 6

Most previous studies on the effects of TKIs on platelet activation have focused on how TKIs affect the GPVI‐mediated activation pathway (here induced by activation with CRP‐XL). However, to get a more complete picture, we also activated platelets with ADP and PAR‐APs (PAR1‐AP and PAR4‐AP) in the presence of TKIs, to ensure we did not miss any potential effects related to other activation pathways. Interestingly, dasatinib and ponatinib treatment resulted in a significant decrease in fibrinogen receptor activation in response to ADP and PAR1‐AP + PAR4‐AP stimulation, respectively (Figure 5F,E). Bosutinib had opposite effects, with a significant trend toward decreased alpha‐granule release but increased lysosomal release, in response to PAR‐APs and ADP, respectively (Figure 5A,D). Nilotinib produced a decrease in P‐selectin expression when platelets were activated with PAR‐AP (Figure 5A). To visualize the interindividual differences, Figures S1‐S5 shows the data for Figures 1, 2, 3, 4, 5 with lines connecting results from the same donor.

### 3.6 EX VIVO platelet function analysis in CML patients

To relate these findings to the clinical context, we also analyzed blood samples from CML patients treated with different TKIs. Figure S7 compares the results from two patients, one treated with bosutinib and one with dasatinib, and shows that the bosutinib‐treated patient exhibits preserved collagen‐induced platelet aggregation and formation of procoagulant PS‐positive platelets (Figure S7A), while both these properties are strongly reduced in the dasatinib‐treated patient (Figure S7B). Figure S8 compares two patients treated with dasatinib, where the one with the higher dose (80 mg/day) shows much less effects on procoagulant subpopulation formation and on coagulation as measured by FOR (Figure S8A) than another patient treated with a lower dose (70 mg/day), where coagulation was much delayed and coagulum elasticity and procoagulant platelet formation were virtually absent (Figure S8B).

### 3.7 Platelet TKI sensitivity map

**Figure 6:**
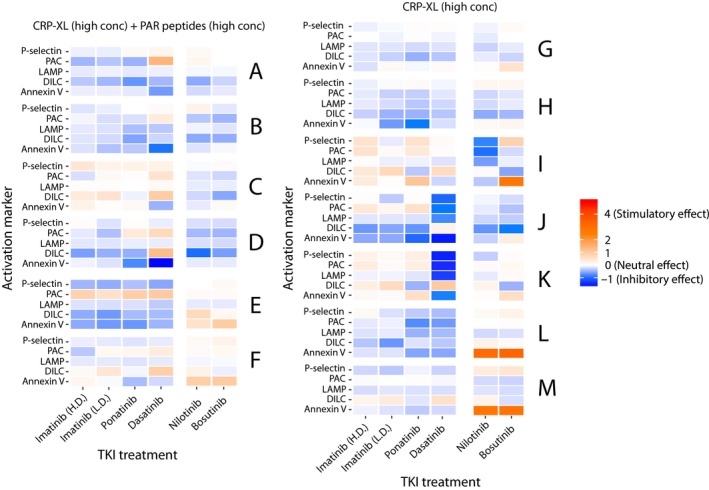
Interindividual variation in the effects of different TKI inhibitors on platelet function. Changes in the platelet response to activation with different combinations of agonists (CRP‐XL 1.2 μg/mL, PAR1‐AP 30 μmol/L, PAR4‐AP 300 μmol/L, ADP 10 μmol/L) in the presence of TKIs (bosutinib, dasatinib, imatinib (low dose), imatinib (high dose), nilotinib, and ponatinib) were measured in healthy donors (n = 7). Results are normalized and rescaled so that 0 denotes the average change in response with all TKIs (ie, 4% inhibition), and the deviation from the mean is denoted in standard deviations (SD) from the average effect for all treatments. Results are visualized so that red represents a stimulatory effect and blue represents an inhibitory effect of the respective TKI

Although some of the TKIs had statistically significant effects on different platelet activation markers in the above group‐wise analysis, we also found notable interindividual variations between the donors. This is illustrated in the line graphs shown in Figure S1‐S5, but these only give information on one hemostatic function at a time, making it difficult to get an overall picture regarding a specific individual's response pattern. Therefore, to enable quantification and visual analysis of these individual differences in a platelet TKI sensitivity map, we calculated the difference (expressed in standard deviations) between an individual test result and the mean effect of all TKIs across the whole panel of agonists and platelet activation markers. This large data set was then visualized in a heat map, enabling a visual comparison of interindividual differences in the effects of the different TKIs for all of the tested donors (Figure 6 and Figure S9). As shown in Figure 6, dasatinib treatment had a strong inhibitory effect on platelet activation after stimulation with a high concentration of CRP‐XL for donors J and K, whereas this effect was much less pronounced for the other donors tested.

## 4 DISCUSSION

Although previous studies have shown that TKIs can affect platelet aggregation,^2^, ^15^, ^46^ the present study represents the first attempt to directly compare the effects of different TKIs when used in clinically relevant drug concentrations on several different aspects of platelet function. This multidimensional approach could prove more informative than conventional assays such as aggregometry‐based platelet function tests, as platelets contribute to hemostasis by activating several important pro‐hemostatic functions that are differentially regulated. Indeed, in a recent study, no correlation was found between decreased platelet aggregation and bleeding symptoms in a cohort of CML patients treated with imatinib, dasatinib, or nilotinib,^47^, ^48^ plausibly due to the lack of tests measuring other aspects of platelet function such as granule secretion and procoagulant activity. As a confirmation of this hypothesis, we found that different TKIs affect platelet function in distinct ways. When investigating features connected to the procoagulant activity of platelets, that is, formation of a PS‐positive platelet subpopulation with depolarized mitochondrial membranes upon strong agonist activation, we found that imatinib did not affect platelet subpopulation formation. However, dasatinib was found to inhibit the formation of procoagulant platelets exposing PS and the mitochondrial membrane depolarization (Figures 1 and 2; Figures S1 and S2). This result agrees with a previous report, where it was found that dasatinib can inhibit platelet function by mechanisms involving inhibition of SRC family kinases (SFK) and immunoreceptor tyrosine‐based activation motif (ITAM) signaling.^49^ In platelets, SFK and ITAM are downstream mediators of the GPVI (collagen) receptor pathway.^50^ The reported association between dasatinib treatment and gastrointestinal bleeding^11^, ^13^ might be related to this suppression of procoagulant platelet formation, which we found to be one of the most affected functions (Figure 1, Figure S1), and which is strongly connected to activation of the GPVI pathway.^35^

In contrast to dasatinib, bosutinib generally induced increased procoagulant platelet formation (Figures 1 and 2; Figure S1 and S2). Our data from the ex vivo study on blood from CML patients treated with TKIs indicate that bosutinib treatment induces stronger platelet aggregation and PS exposure than dasatinib treatment (Figure S7). To the best of our knowledge, there are, to date, no reports on the effects of bosutinib (or nilotinib) on platelet signaling. Previously, bosutinib has been reported to have fewer and milder off‐target activities.^3^ However, bosutinib can upregulate mitogen‐activated protein kinase (MAPK) in cancer cells.^51^ In platelets, MAPK is directly linked to GPVI‐mediated signaling.^52^ Increased activation of MAPK might explain why bosutinib treatment leads to more procoagulant platelet formation.^53^

When investigating other features of platelet activation, such as granule release and activation of the fibrinogen receptor, we found that treatment with dasatinib decreased the platelet granule release (Figure 3, Figure S3) and activation of GPIIb/IIIa as a response to CRP‐XL (Figure 4A, Figure S4A). This finding was quite expected, as earlier findings have shown that dasatinib effectively inhibits platelet function.^46^ The increase in GPIIb/IIIa activation seen with combined treatment with CRP‐XL + PAR‐APs was also expected as a consequence of the inhibition of the formation of the procoagulant platelet subpopulation by dasatinib, as procoagulant platelets normally show reduced binding of PAC‐1 (Figure 4B, Figure S4B).^35^ In situations where stimulation was insufficiently strong to result in procoagulant platelet formation, as with ADP, dasatinib treatment resulted in a decrease in PAC‐1 binding. A similar effect was also found for treatment with ponatinib and bosutinib.

We have already discussed that, on a group basis, each TKI showed a specific pattern of effects on platelets. But whether those TKI‐specific effects will be similar in all treated individuals or not is an important and clinically relevant question. According to our results, the answer is no. We found marked interindividual variation in vitro in response to the different TKIs, especially to dasatinib, nilotinib, ponatinib, and bosutinib. This observation is also supported by our clinical data where we observed notable interindividual differences in platelet responses in dasatinib‐treated patients (Figure S8). This may help to explain why some patients on dasatinib therapy are more prone to bleeding than others and why patients treated with bosutinib, nilotinib, and ponatinib have a higher risk of cardiovascular events. Therefore, it might be important to find out who will be more prone to altered hemostasis during TKI therapy. To visually compare the effects of the different TKIs on platelet functions for different individuals, we therefore developed a platelet TKI sensitivity map (Figure 6, Figure S9). By performing platelet function tests on blood samples from patients using different TKIs, it might be possible to test and visualize the susceptibility of the individuals to treatment‐induced platelet dysfunction. For example, it can be clearly visualized from the color index of the sensitivity map that although treatment with dasatinib had inhibitory effects (blue color) for most activation markers after stimulation with CRP‐XL, these effects were much more pronounced for the donors J and K than for the other donors, whereas bosutinib and nilotinib had marked stimulatory effects on procoagulant activity (shades of red for annexin V) after stimulation with CRP‐XL for donors L and M but much weaker effects for the other donors (Figure 6). Ponatinib, on the other hand, stimulated P‐selectin exposure (alpha‐granule secretion) and LAMP‐1 (lysosomal exocytosis) after stimulation with CRP‐XL in low concentrations for donors M, N, and Q.

In conclusion, when conducting a group‐wise comparison, we found that dasatinib generally inhibited platelet functions, whereas bosutinib had stimulatory effects on procoagulant platelet formation. However, as we observed notable and potentially relevant interindividual differences in response to TKIs, tools for assessment of idiosyncratic effects of these drugs such as the proposed sensitivity map could prove valuable for individualization of treatment and avoid side effects related to platelet dysfunction in the individual patient.

## AUTHOR CONTRIBUTIONS

SD: Concept, experiments, data analysis, and writing; NB: Concept, design, data analysis, and writing; CS: Experiments and analysis; AT: Experiments and analysis; SR: Design, materials, data analysis, and writing; KL: Design, patient samples, material, and writing.

## Data Availability

The data that support the findings of this study are available from the corresponding author upon reasonable request.
