# Supplementary material for: Varying effects of tyrosine kinase inhibitors on platelet function—A need for individualized CML treatment to minimize the risk for hemostatic and thrombotic complications?
Source: Cancer Med. 2019 Nov 12;9(1):313–23. doi: 10.1002/cam4.2687 (PMC6943147; doi:10.1002/cam4.2687)
Supplement: Supplementary file 1 [file CAM4-9-313-s001.doc]

**Supporting Information**

**For**

**Varying effects of tyrosine kinase inhibitors on platelet function – a need for individualized CML treatment to minimize the risk for hemostatic and thrombotic complications?**


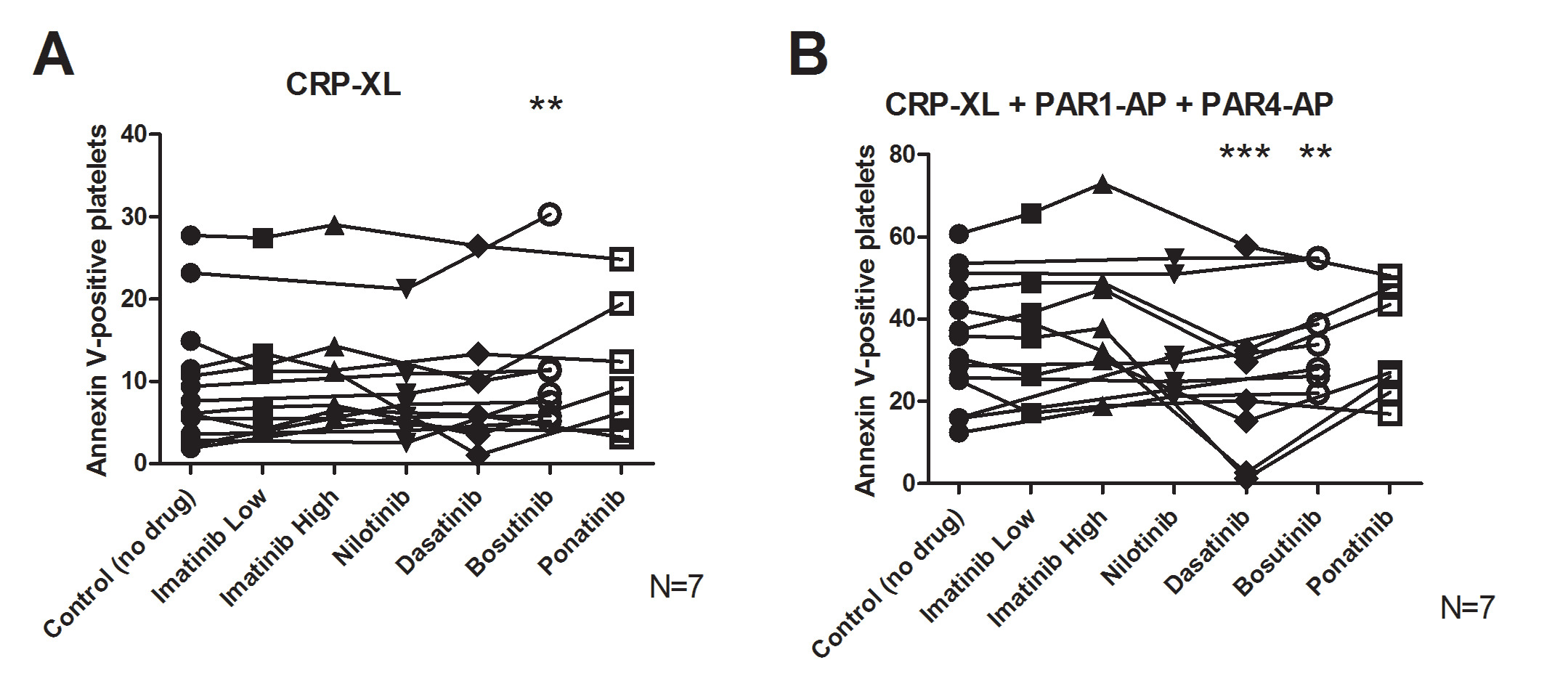


*Figure S1: Scatter plots with connecting lines for each individual donor which corresponds to figure 1 (Changes in pro-coagulant membrane exposure in presence of TKIs) in the main manuscript text.*


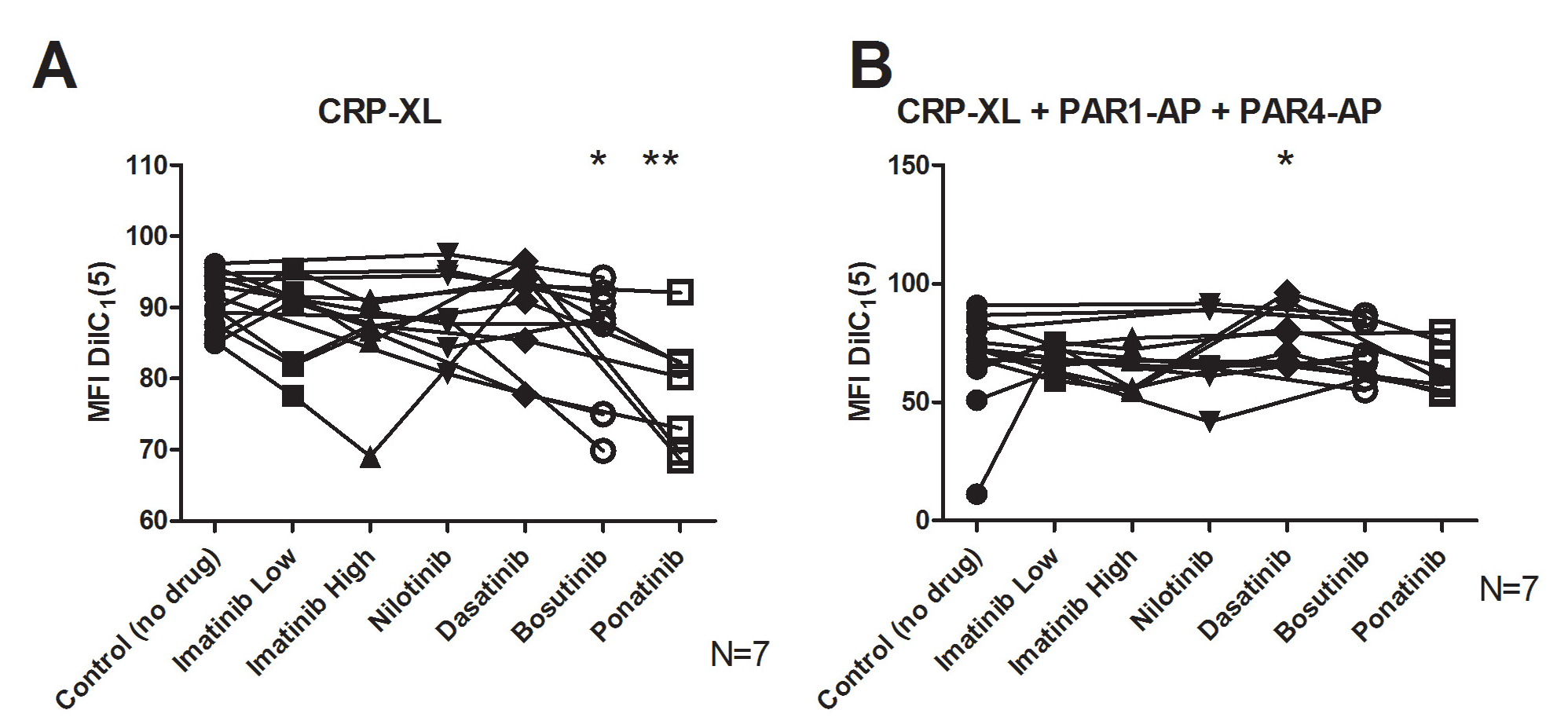


*Figure S2: Scatter plots with connecting lines for each individual donor which corresponds to figure 2 (Changes in mitochondrial membrane potential in presence of TKIs) in the main manuscript text.*


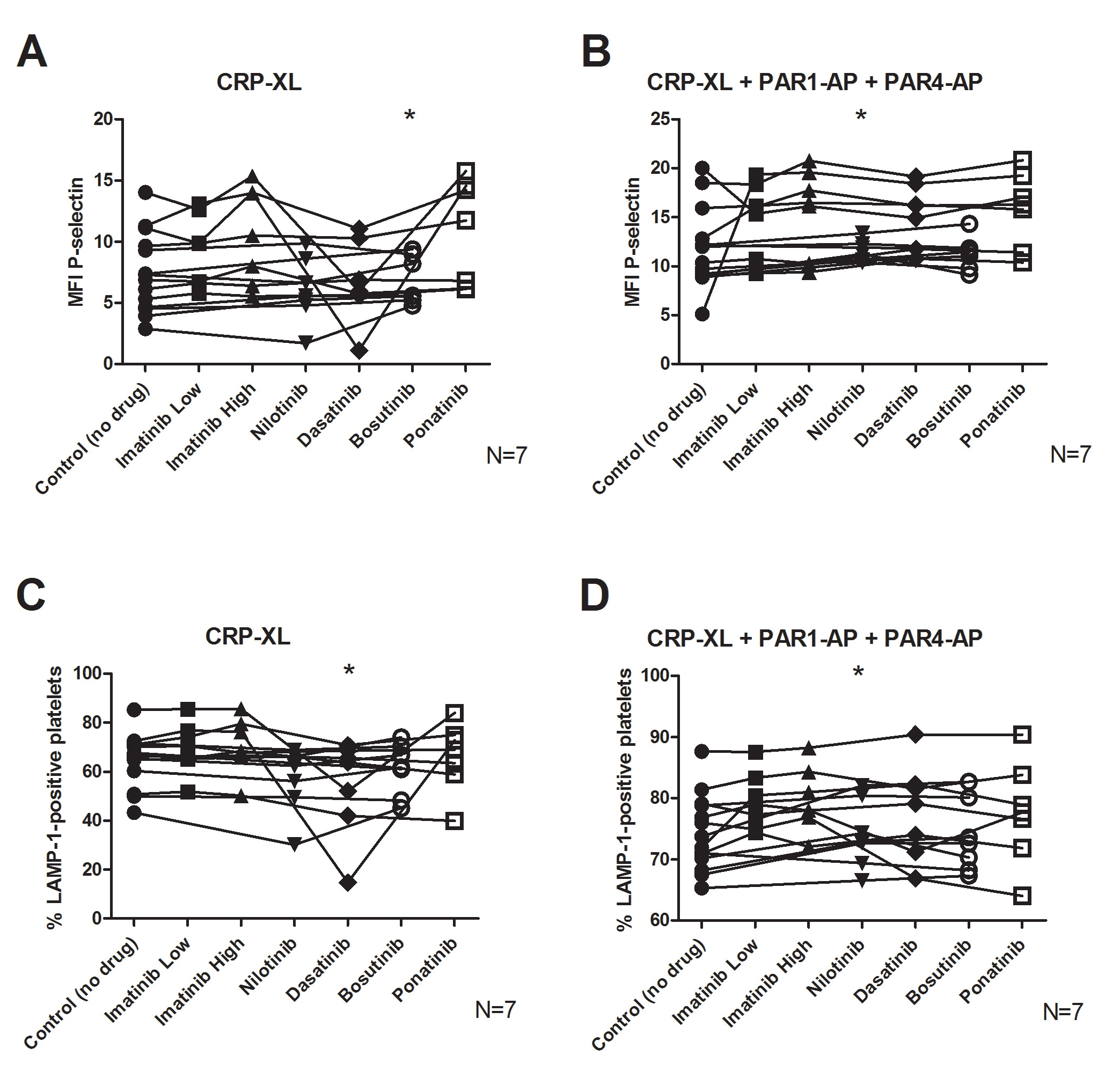


*Figure S3: Scatter plots with connecting lines for each individual donor which corresponds to figure 3 (Alteration of degranulation potential by TKIs) in the main manuscript text.*


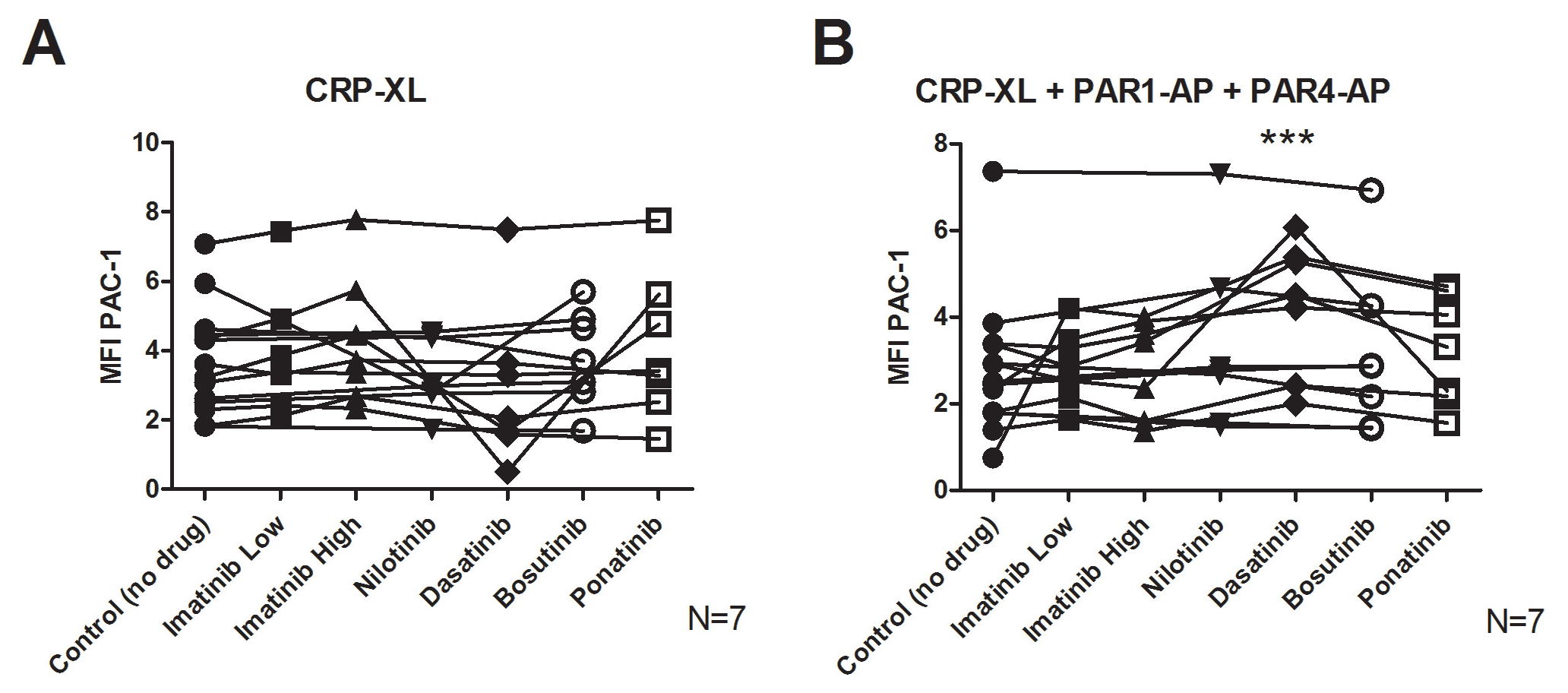


*Figure S4: Scatter plots with connecting lines for each individual donor which corresponds to figure 4 (Alteration of platelet fibrinogen receptor activation by TKIs) in the main manuscript text.*


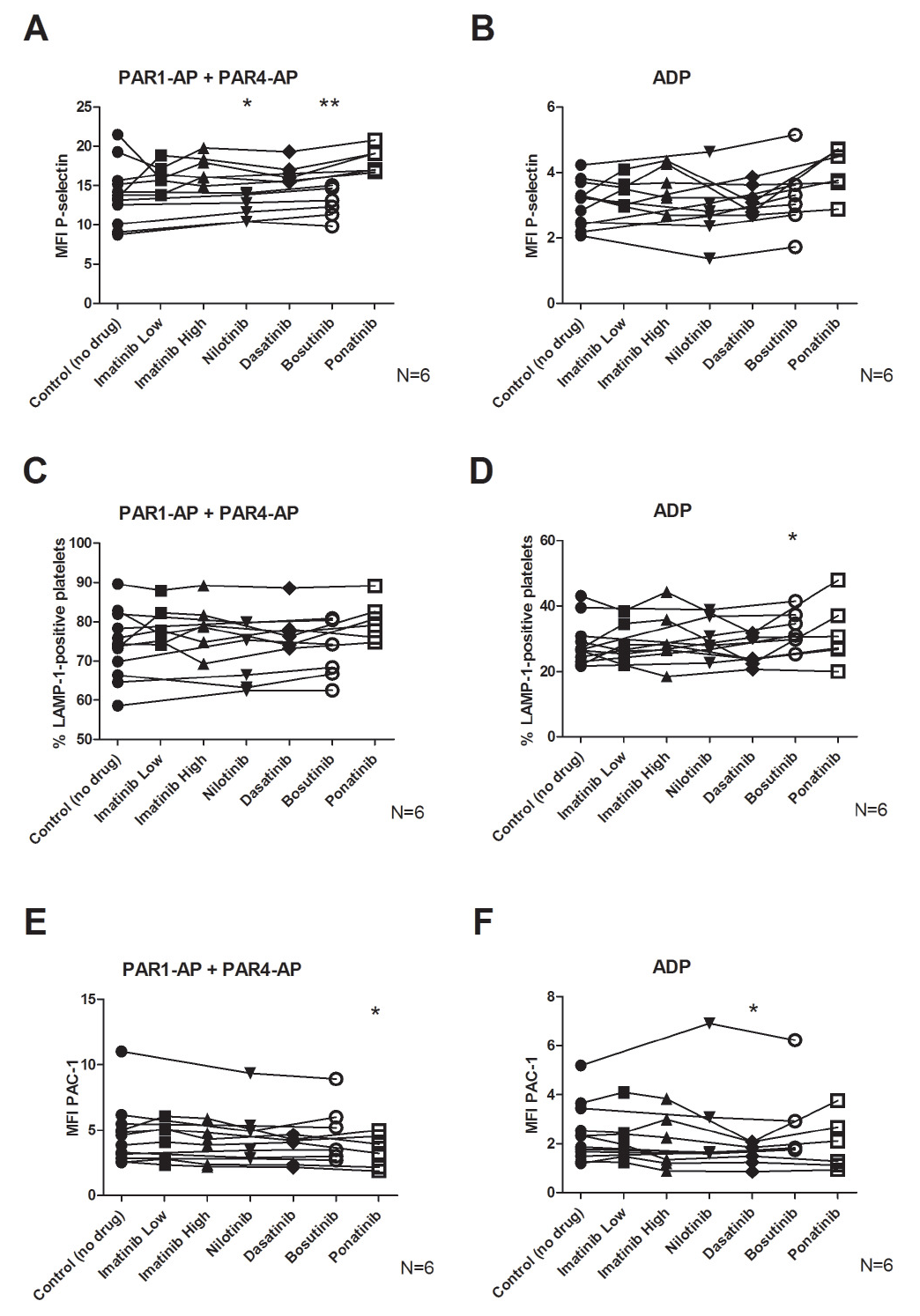


*Figure S5: Scatter plots with connecting lines for each individual donor which corresponds to figure 5 (Alteration of platelet function by TKIs when activated with PAR-APs and ADP) in the main manuscript text.*

*
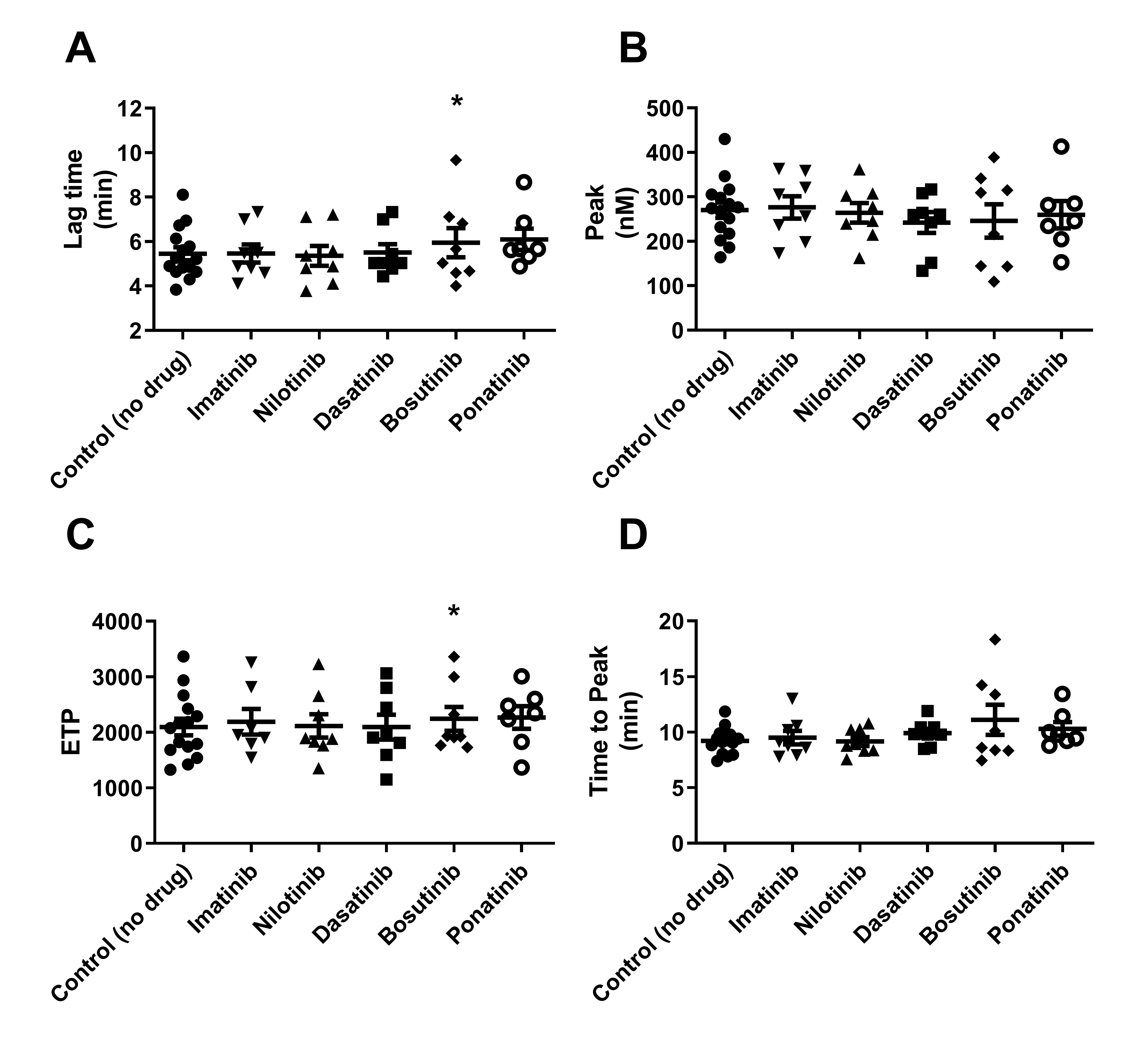
*

*Figure S6: Alteration of thrombin generation in presence of different TKIs. Platelet-rich plasma with TKIs was first incubated with CRP-XL (1.2μg/ml) before the addition of calcium and tissue factor to start thrombin generation. The graphs show changes induced by different TKIs on the kinetics of thrombin production; (A) the Lag time (the time between tissue factor addition and start of thrombin generation), (B) the peak height, (C) ETP (endogenous thrombin potential) and (D) the time to reach the peak thrombin concentration. The scatter plots show results for the individual donors, the mean value and standard error of the mean (SEM) are also shown. Paired raw data have been used for ANOVA testing. Stars (*) denote significant differences from control where *= P<0.05, n=7 to 8. Imatinib was only tested in the lower dose in these experiments.*

**Dasatinib vs bosutinib (clinical ex vivo data):**

A bosutinib-treated CML patient showed higher platelet aggregation than a dasatinib-treated patient (especially when collagen was used as agonist). The percentage of procoagulant (annexin V-positive) platelets was also higher in the bosutinib-treated patient.


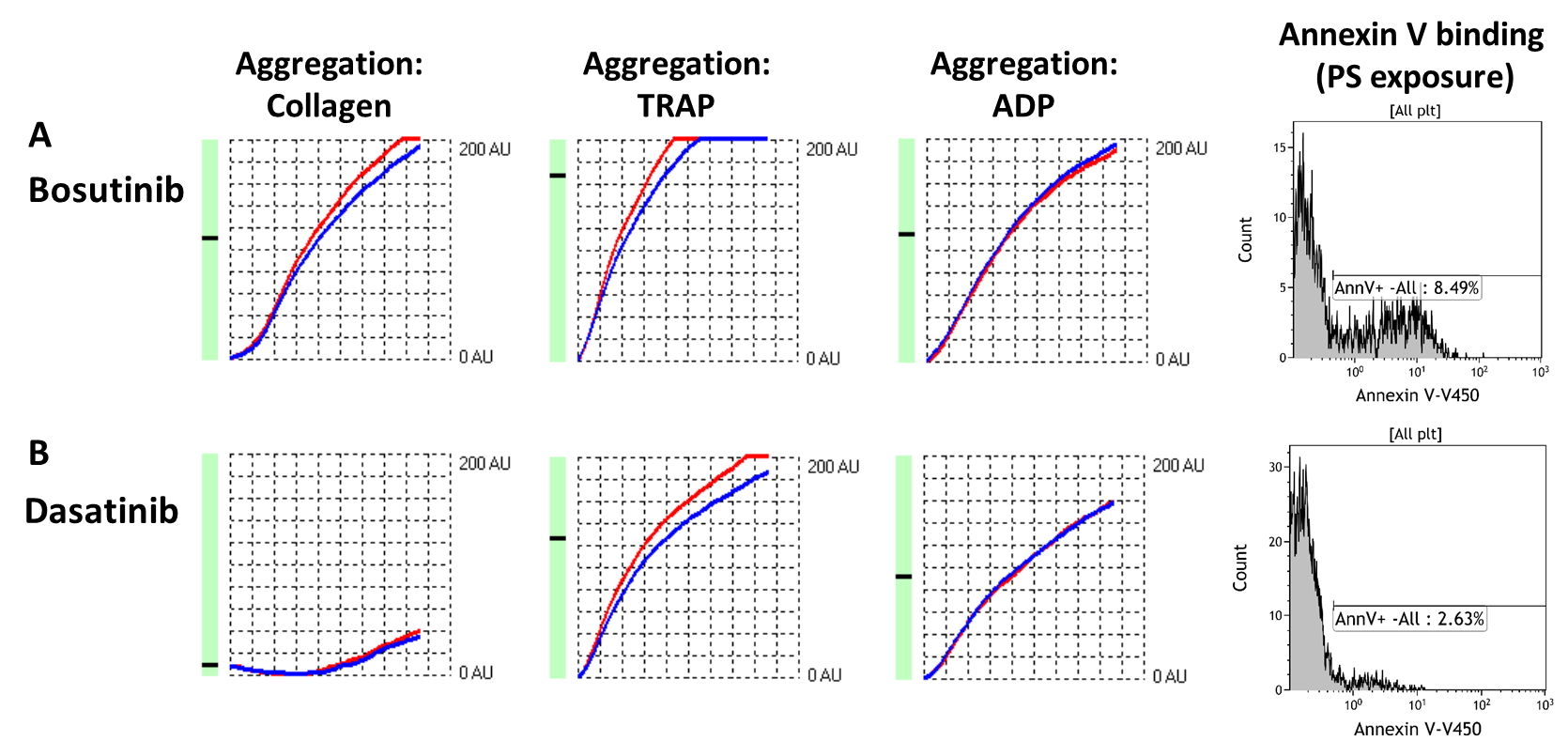


*Figure S7:* **(A)** Bosutinib-treated patient showing high collagen-induced aggregation in whole blood aggregometry (Multiplate® using the commercially available COLtest (collagen), TRAPtest (TRAP=PAR1-AP) and ADPtest from the same manufacturer) and PS exposure (i.e. binding of annexin V) in platelets activated with CRP-XL (1.2μg/ml) + PAR-APs (PAR1- (30μM) and PAR4-AP (300μM)). **(B)** Dasatinib-treated patient showing reduced aggregation in response to collagen and almost no PS exposure in platelets activated with CRP-XL (1.2μg/ml) + PAR-APs (PAR1- (30μM) and PAR4-AP (300μM)). Blue and red lines in the aggregation plots represents the two measurements performed in each test cell. Flow cytometry data showing results for the total platelet population (All platelets).

**Inter-individual variation in dasatinib-treated patients (clinical ex vivo data):**

Inter-individual variation in TKI effects on hemostasis was also present among CML patients. A patient treated with dasatinib 80mg/day (Figure S2A) showed higher PS exposure, more rapid coagulation and better clot elasticity development than a patient treated with dasatinib 70mg/day (Figure S2B).


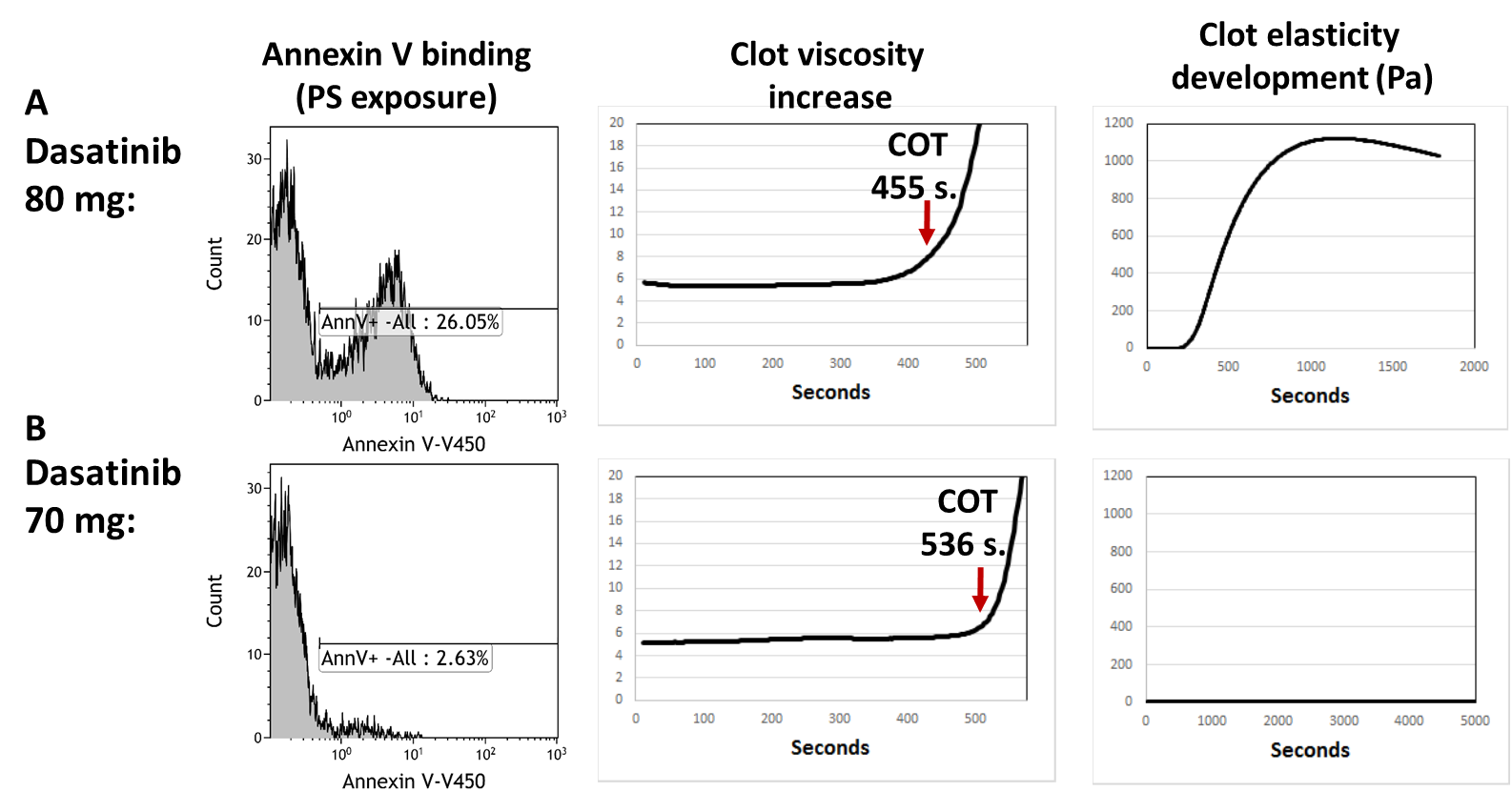


*Figure S8*: **(A)** Dasatinib (80mg/day)-treated patient showing PS exposure (i.e. binding of annexin V) when activated with CRP-XL (1.2μg/ml) + PAR-APs (PAR1-AP (30μM) and PAR4-AP (300μM)), and in vitro thrombus formation with coagulation (COT=clot onset time) and clot elasticity (represents clot strength) as detected by free oscillation rheometry (ReoRox G2). **(B)** Dasatinib (70mg/day)-treated patient showing no PS exposure, a prolonged COT (red arrow) and no clot elasticity development.


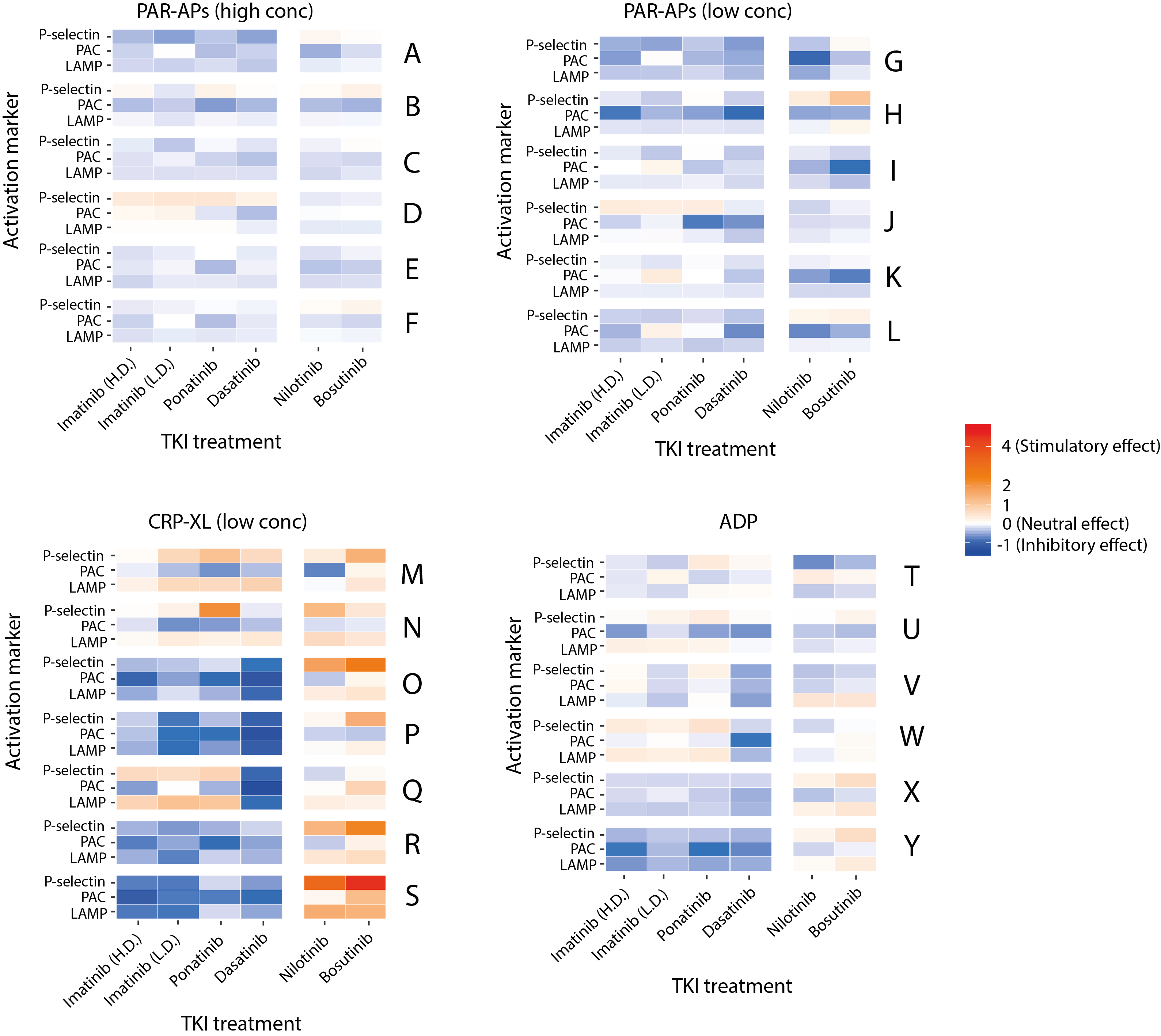


*Figure S9: Inter-individual variation in the effects of different TKI inhibitors on platelet function.* Changes in the platelet response to activation with different combinations of agonists (CRP-XL high conc. (1.2μg/ml), CRP-XL low conc. (0.0375μg/ml), PAR1-AP high conc. (30μM), PAR1-AP low conc. (5μM), PAR4-AP high conc. (300μM), PAR4-AP low conc. (50μM), ADP (10μM)) in the presence of TKIs (bosutinib, dasatinib, imatinib (low dose), imatinib (high dose), nilotinib and ponatinib) were measured in healthy donors (n=7). Results were normalized and rescaled so that 0 denotes the average change in response with all TKIs (i.e. 4 % inhibition), and the deviation from the mean is denoted in standard deviations (S.D.) from the average effect. Results are visualized so that red represents a stimulatory effect and blue represents an inhibitory effect of the respective TKI.
